# Supplementary material for: Subsynaptic Distribution, Lipid Raft Targeting and G Protein-Dependent Signalling of the Type 1 Cannabinoid Receptor in Synaptosomes from the Mouse Hippocampus and Frontal Cortex
Source: Molecules. 2021 Nov 16;26(22):6897. doi: 10.3390/molecules26226897 (PMC8621520; doi:10.3390/molecules26226897)
Supplement: Supplementary file 1 [file molecules-26-06897-s001.zip › molecules-1441999-supplementary-edit-clear.pdf]

molecules

Supplementary Information

Subsynaptic Distribution, Lipid Raft Targeting and G Protein-Dependent Signalling of the Type 1 Cannabinoid Receptor in Synaptosomes from the Mouse Hippocampus and Frontal Cortex

Miquel Saumell-Esnaola [1,2,3], Sergio Barrondo [1,2,4], Gontzal García del Caño [2,5], María Aranzazu Goicolea [6], Joan Sallés [1,2,4], Beat Lutz [3], and Krisztina Monory [3,*]

[1] Department of Pharmacology, Faculty of Pharmacy, University of the Basque Country UPV/EHU, 01006 Vitoria-Gasteiz, Spain; miquel.saumell@ehu.eus (M.S.-E.); sergio.barrondo@ehu.eus (S.B.); joan.salles@ehu.eus (J.S.)
[2] Bioaraba, Neurofarmacología Celular y Molecular, 01009 Vitoria-Gasteiz, Spain; gontzal.garcia@ehu.eus
[3] Institute of Physiological Chemistry, University Medical Center of the Johannes Gutenberg University Mainz, 55128 Mainz, Germany; beat.lutz@uni-mainz.de
[4] Centro de Investigación Biomédica en Red de Salud Mental (CIBERSAM), 28029 Madrid, Spain
[5] Department of Neurosciences, Faculty of Pharmacy, University of the Basque Country UPV/EHU, 01006 Vitoria-Gasteiz, Spain
[6] Department of Analytical Chemistry, Faculty of Pharmacy, University of the Basque Country UPV/EHU, 01006 Vitoria-Gasteiz, Spain; mariaaranzazu.goicolea@ehu.eus
* Correspondence: monory@uni-mainz.de; Tel.: +49-6131-39-24551

1. Supplementary materials and methods

1.1. Chemicals and Antibodies

BCA Protein Quantification Kit was purchased from Abcam (Cambridge, UK). Bio-Rad Protein Assay Dye Reagent Concentrate was purchased from Bio-Rad (Hercules, California). Calbiochem Cholesterol/Cholesteryl Ester Quantitation Kit and Guanosine 5′-o-(3-[35S]thio-triphosphate) ([35S]GTPγS, 1000-1400 Ci/mmol) were purchased from Perkin-Elmer (Barcelona, Spain). Protease inhibitor cocktail (phenylmethylsulfonyl fluoride - PMSF- and iodoacetamide), GDP, GTP, GTP S, WIN 55,212-2, and methyl-β-cyclodextrin were purchased from Sigma Chemical (St. Louis, MO, USA). CP 55,940 was purchased from Tocris BioScience (Minneapolis, MN, USA). DiIC16 dye was purchased from Invitrogen (Waltham, MA, USA). PNGase F was purchased from New England Biolabs (Ipswich, MA, USA).

Supplementary Table S1 contains the list of the primary antibodies used. Secondary antibodies for Immunofluorescence and Western blot were Alexa Fluor 488 Goat anti-Rabbit (Invitrogen, A11034), DyLight 649 Donkey anti-Mouse (Jackson Immuno Research, 715-496-151), anti-Rabbit IgG HRP conjugate (Amersham, NA934), anti-Mouse IgG HRP conjugate (Amersham, NXA931), and anti-Goat IgG HRP conjugate (Sigma-Aldrich, A5420).

1.2. Immunofluorescence assay for frontal cortex synaptosomes

Synaptosomes from mouse frontal cortex were thoroughly re-suspended by pipetting and vortexing in 0.5% DMSO containing 0.1 M phosphate buffer (PB) to achieve a total protein concentration of 50 μg/150 μL. The synaptosomal suspension (2.5 mL) was first passed through a 20 μm filter and then through a 5 μm filter. The filtrate was then 1:1

diluted with 2.5 mL 0.5% DMSO containing PB, which was passed through the 5 μm filter. Then, 150 μL of filtered synaptosomes suspension (corresponding to 25 μg synaptosomal protein at origin) was seeded onto 18 mm round poly-L-ornithine covered coverslips placed on parafilm-covered glass platform on ice for 1 h. After seeding, the buffer was aspirated, and coverslips were washed with cold PBS to remove unattached particles. Then, the synaptosomes were incubated with DiIC16 at room temperature for 10 min. Before washing three times quickly with PBS, synaptosomes were fixed with phosphate-buffered paraformaldehyde 4% for 15 min. After washing three times quickly with PBS, synaptosomes were permeabilized with Triton X-100 0.05% in PBS for 5 min. Thereafter, synaptosomes were washed quickly with PBS and proceed with the immunofluorescence assay. Synaptosomes were blocked with non-Perm buffer with 1% BSA (w/v) and 1% Normal Serum from the species in which the secondary antibody was raised for 30 min. Subsequently, synaptosomes were incubated overnight at 4 °C with the primary antibody at the appropriate dilution (see Table S1 for details), washed three times with Non-Perm buffer (10 min each wash), and incubated for 1 h at room temperature in an adequate dilution of the fluorescent dye-conjugated secondary antibody. Finally, synaptosomes were washed three times with Non-Perm buffer (10 min each) and mounted with Mowiol immediately. Fluorescent images were captured with an epifluorescence microscope Carl Zeiss Axio Observer.Z1, equipped with a HXP 120 C metal halide lamp illumination source, a high-resolution monochromatic camera (AxioCam MRm, 1388 9 1040 pixels), a structured illumination module (ApoTome), and a XYZ motorized stage (all from Carl Zeiss MicroImaging, Inc, Gottigen, Germany). Images were taken using a 63 9 Plan-Apochromat objective (NA 1.4). Bandpass filters used were 49 DAPI (Ex G 365/Em 445/50), 38 HE eGFP (Ex 470/40, Em 525/50), 43 HE Cy3 shift free (Ex 550/25, Em 605/70), and DyLight 549. Images were digitized using Zeiss Axio Vision 4.8 software (Carl Zeiss MicroImaging, Inc). For illustration, images were exported to TIFF format and were compiled and labeled using Adobe Photoshop CS3 (San Jose, CA, USA). ImageJ image analysis software (NIH, Bethesda, MD, USA) was used for the quantification of the size and the neural origin of the particles present in the synaptosomal enriched fraction. After assigning a pseudocolor to each channel and despeckling, the images were binarized and analyzed by specific analysis plugins. GraphPad Prism (version 5.0, GraphPad Software Inc., San Diego, CA, USA) was used to organize data. Results are expressed as mean ± standard error (SEM), performing at least two independent experiments.

1.3. Western blot assay in purified fractions of synaptosomal membranes

Briefly, samples were boiled for 10 min in urea-denaturing buffer (20 mM Tris-HCl, pH 8.0, 12% glycerol, 12% Urea, 5% dithiothreitol, 2% sodium dodecyl sulfate (SDS), 0.01% bromophenol blue). Denaturized proteins were resolved by electrophoresis on SDS–polyacrylamide (SDS–PAGE) gels using the Mini Protean II gel apparatus (Bio-Rad, Hercules, CA, USA) for about 2 h at 100 V and transferred to 100% methanol-activated polyvinylidene fluoride (PVDF) membranes using the Mini TransBlot transfer unit (Bio-Rad) at 30 V constant voltage overnight at 4 °C. Thereafter, PVDF membranes were briefly washed with distilled H2O and allowed to dry and re-activated in 100% methanol. After washing 10 min in washing buffer (PBS with 0.1% Tween-20) at room temperature, PVDF membranes were incubated in blocking buffer (PBS with 0.2% Tween-20, 5% non-fat dry milk and 0.5% BSA) for 1 h at room temperature, followed by overnight incubation at 4 °C with the corresponding primary antibodies diluted in blocking buffer at the adequate concentrations (see Table S1). Blots were washed and incubated with specific horseradish peroxidase conjugated secondary antibodies diluted (1:10,000) in blocking buffer for 1.5 h at room temperature. Immunoreactive bands were revealed with the ECL system according to the manufacturer instructions, either developed on X-ray films or acquired using an ImageQuant 350 digital image system (GE Healthcare). Digital images of immunoreactive bands, either from X-ray films using a transmittance scanner or acquired using the Im-

ageQuant 350 imager device, were quantified by densitometry using ImageJ image analysis software (ImageJ, NIH, Bethesda, MD, USA). For comparison of protein expression between samples, a specific method of semi quantitative analysis was chosen depending on the availability of protein level of samples. The most accurate method used was the comparison of slopes. Plotting increasing amount of protein and integrated optical density (OD) values of immmunoreactive signals obtained for each total protein loading allowed us to obtain a linear regression equation for each sample. The ratio between the slopes of the curves allowed us to determine the fold of change on the expression of the protein analyzed. When the total amount of protein was limited, the semi quantitative analysis of protein expression was performed constructing a standard curve and integrating the OD values of experimental samples in the standard curve equation or directly calculating the ratio between OD values of experimental samples bands after resolving equal amounts of total protein. The total protein loading was checked using antibodies against proteins enriched in synaptosomes or by Coomassie Brilliant Blue gel staining method. GraphPad Prism (version 5.0, GraphPad Software Inc., San Diego, CA, USA) was used to organize and statistically analyze data. Results were expressed as a ratio between the slopes of the curves or as mean ± standard error (SEM), performing at least three independent experiments. The statistical analysis is detailed in each figure legend, which was carried out using two-way ANOVA followed by sidak test or by regression curves analysis comparing slopes. Statistical significance was set at the 95% confidence level.

1.4. Agonist stimulated [^{35}S]GTPγS binding assay in synaptosomal membranes

[^{35}S]GTPγS binding assays were performed with 5 μg of total protein of mouse synaptosomal membrane per tube, which was incubated at 30 °C for 2 h in [^{35}S]GTPγS-incubation buffer (0.5 nM [^{35}S]GTPγS, 1 mM EGTA, 3 mM $MgCl_2$, 100 mM NaCl, 0.2 mM DTT, 50 μM GDP, 0.5% BSA fatty acid free, and 50 mM Tris-HCl, pH 7.4). The CB1 cannabinoid receptor agonist CP 55,940 or WIN 22,12-2 (1 nM - 30 μM) was added to determine receptor-stimulated [^{35}S]GTPγS binding. Nonspecific binding was defined in the presence of 10 μM unlabelled GTPγS. Basal binding was assumed to be the specific [^{35}S]GTPγS binding in the absence of agonist. The reactions were terminated by rapid vacuum and filtration through Whatman GF/B glass fibre filters, and the remaining bound radioactivity was measured by liquid scintillation spectrophotometry.

The concentration-dependent increase in specific binding of [^{35}S]GTPγS elicited by CP 55,940 or WIN 55,212-2 was expressed as a percentage of the basal unstimulated value and analyzed using nonlinear regression with GraphPad Prism (version 5, GraphPad Software; San Diego, CA, USA) to determine the concentration eliciting the half-maximal effect (EC50), maximum percent increase (% Emax), and slope factor. Concentration response curves were constructed using mean values ± SEM from triplicate data points (expressed as specific [^{35}S]GTPγS bound over basal) performing at least three independent experiments. The EC50 values were transformed into pEC50 (−logEC50) to be analyzed, because it has been demonstrated that parameters such as EC50 and affinity constants obtained experimentally are log-normally distributed; therefore, statistical analysis should be performed as such [65,66]. The statistical significance of differences between %Emax and log EC50 values was analyzed by Student's t-test or by one-way ANOVA followed by Sidak or Tukey´s post-test, which is detailed in each figure legend. Statistical significance was set at the 95% confidence level.

2. Supplementary Figures and Figure Legends

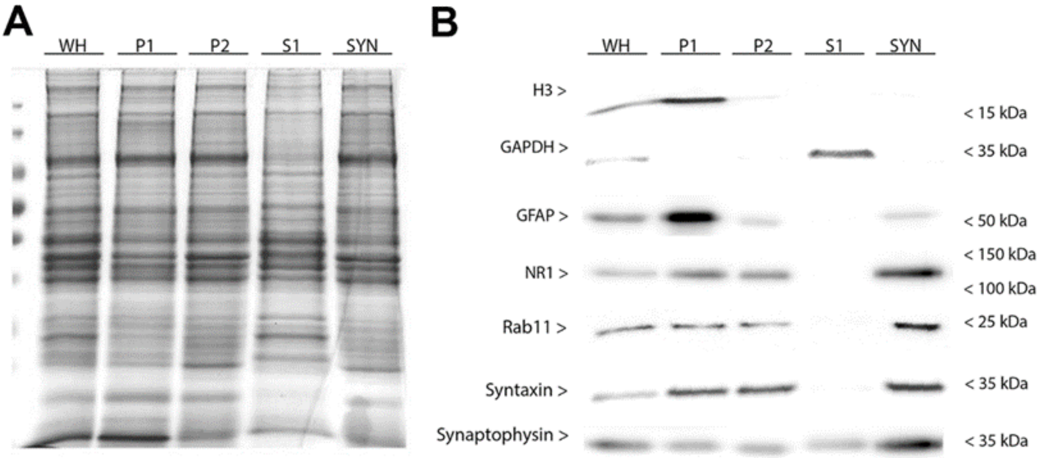

Figure S1. Western blot of the homogenate and subcellular fractions obtained from sequential fractionation of adult mouse brain cortical homogenates. **(A)** SDS-PAGE and Coomassie blue staining of the obtained fractions run in parallel (10 μg/lane). WH, Whole homogenate; P1, crude nuclei; P2, crude membranes/crude synaptosomes; S1, cytoplasm; SYN, synaptosomes. **(B)** Representative images of Western blots performed in the same samples using antibodies as markers of specific sub-cellular compartments. Each antibody staining was originated from different Western blot procedure. Immunoblot against histone 3 (H3), glyceraldehyde-3-phosphate dehydrogenase (GAPDH), glial fibrillary acid protein (GFAP), NMDA receptor subunit 1 (NR1), Ras-related protein 11b (Rab11b), syntaxin 1a and synaptophysin. Protein migration was consistent with their predicted molecular mass (H3, 15.4 kDa; GAPDH, 35.8 kDa; GFAP, 50 kDa; NR1, 130 kDa; Rab11b, 24.5 kDa; Synaptophysin, 34 kDa; Syntaxin 1a, 33 kDa. On the right margin, the migration of molecular weights from the marker is indicated.

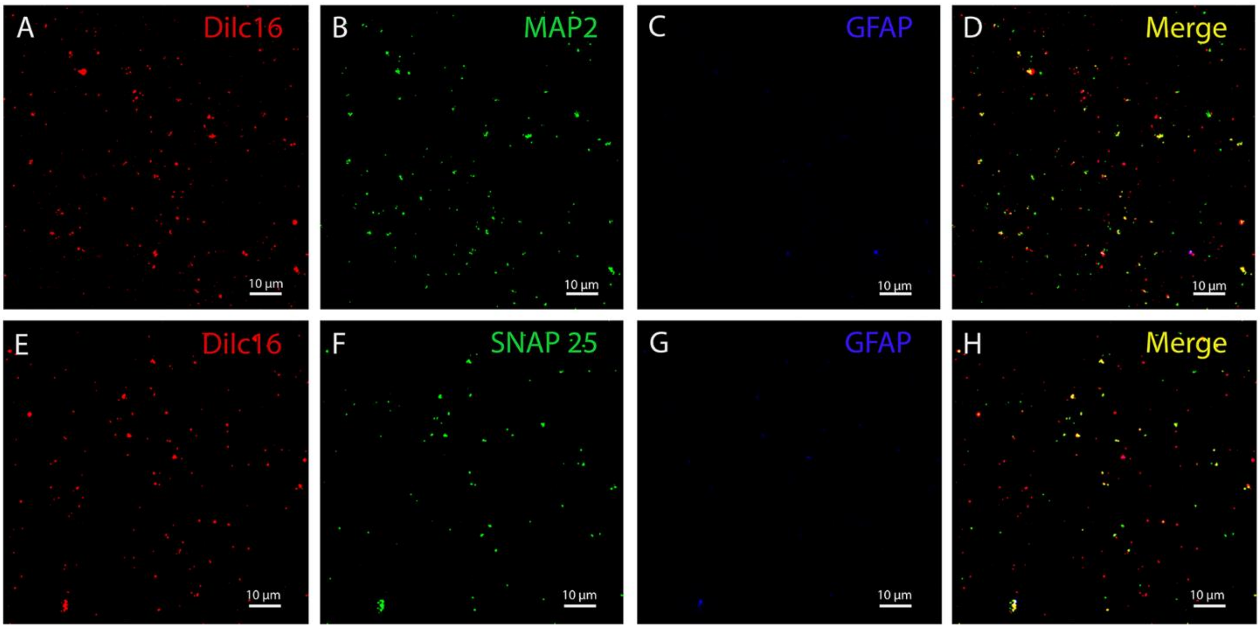

Figure S2. Double-immunofluorescence MAP2/GFAP and SNAP25/GFAP combined with the membrane marker DiIC16 in isolated cortical synaptosomes maintained in isotonic buffer and seeded on poly-L-ornithine coated coverslip. Quantification of particles size. 79.65% ± 2.33% between 0.25-1.5 μm; 6.89% ± 3.40% >1.5 μm; 14.47% ± 0.25% < 0.25 μm. Quantification of particles origin. Positive for MAP2: 48.57% ± 4.54%; SNAP25: 44.75% ± 2.37%; GFAP: 4.64% ± 1.29%; Non-identifiable: 48.69% ± 2.42%. Data values are mean ± SEM. Two independent assays were performed.

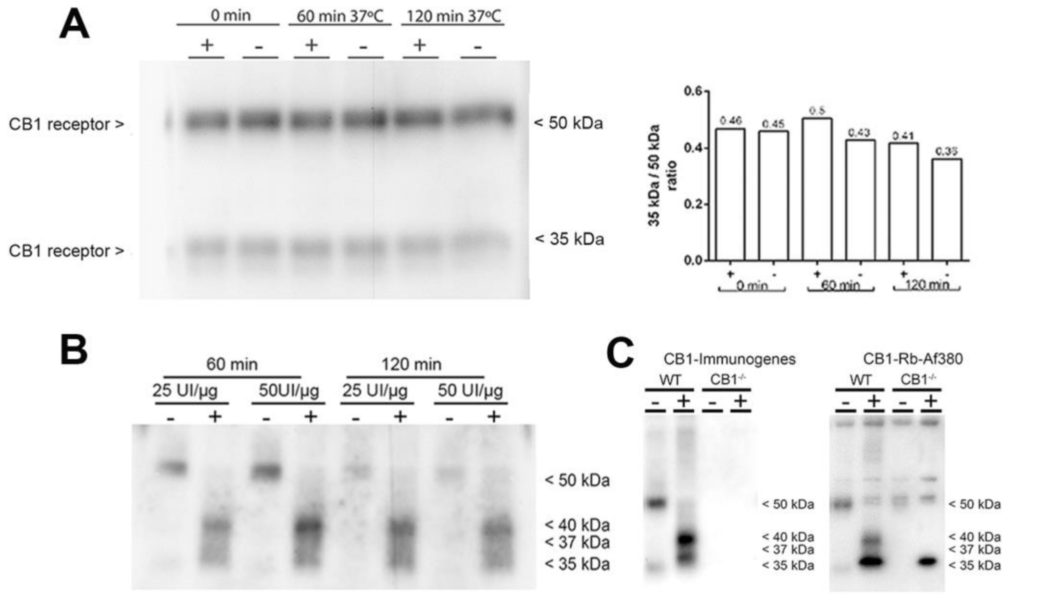

Figure S3. Migration profile of the CB1 receptor immunorreactive bands in synaptosome samples subjected to a potentially proteolytic condition and to an N-glycosidase treatment with the PNGase F enzyme. (A) Representative Western blot carried out loading same amount of total protein of frontal cortical control synaptosomes and of synaptosomes incubated at 37 °C for 1 or 2 hours with or without protease inhibitors (20 µg/lane). Immunoblot against CB1 receptor protein using the antibody from CB1-immunogenes. The molecular weights depicted correspond to the signal of the standard markers. In bar graphs, the ratio between the optical densities of the two immunoreactive bands is indicated. (B) Representative Western blots carried out loading the same amount of frontal cortical control synaptosomes and of N-glycosidase F (25 or 50 UI/µg) treated synaptosomes incubated at 37 °C for 1 or 2 hours. Immunoblot against CB1 receptor protein using the CB1-immunogenes antibody. The approximate molecular masses of the immunoreactive species detected on the blot are indicated. (C) Representative Western blots carried out loading the same amount of control and N-glycosidase F treated (25 UI/ µg total synaptosomal protein; one hour at 37 °C) frontal cortical synaptosomes from WT and CB1-KO mice (20 µg total protein/lane). Immunoblot against CB1 receptor protein using CB1-immunogenes and CB1-Rb-Af380 antibodies. The approximate molecular masses of the immunoreactive species detected on the blot are indicated.

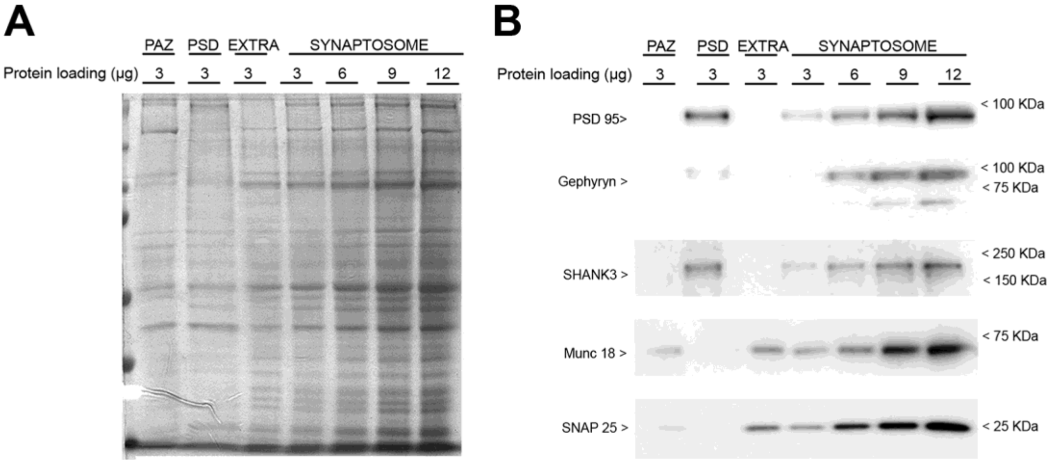

Figure S4. Subsynaptic compartmentalization of the selected markers in PAZ, PSD and EXTRA fractions isolated from cortical synaptosomes derived from wild-type mice. (A) Representative Coomasie blue-stained SDS-PAGE and (B) Western blots carried out by immunoblotting increasing amounts of cortical synaptosomes (3, 6, 9, and 12 µg/lane) and different subsynaptic fractions (3 µg/lane) of wild-type mice. Presynaptic fraction PAZ, postsynaptic fraction PSD, and extrasynaptic fraction EXTRA. The efficiency of the subsynaptic separation was assessed using antibodies

as markers of specific sub-cellular compartments: PSD fraction markers (PSD-95, Gephyrin, and Shank3) and EXTRA and PAZ fractions markers (Munc-18 and SNAP-25). Protein migration was consistent with their expected molecular mass, except for PSD-95 protein (PSD- 95, 80.4 kDa; Gephyrin, 83.2 kDa; Shank3, 185.3 kDa; Munc-18, 66.3 kDa; SNAP-25, 23.3 kDa). The molecular weights depicted correspond to the sizes of the standard markers.

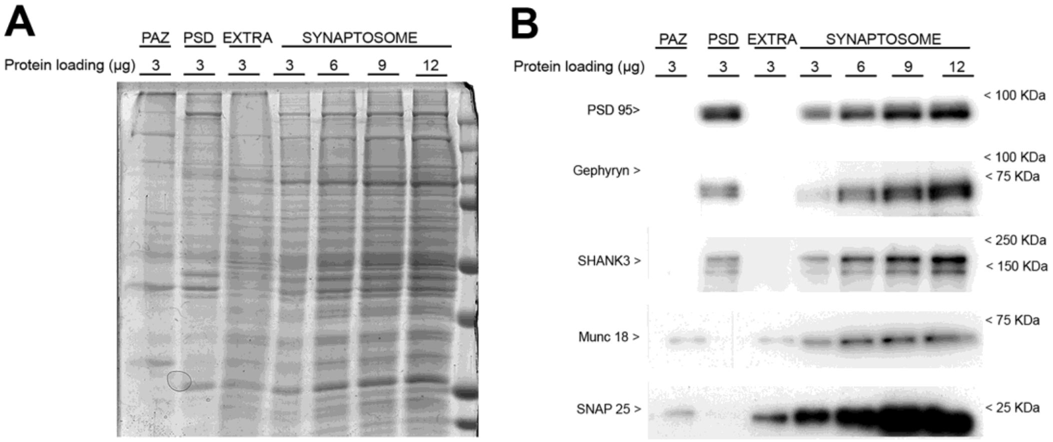

Figure S5. Subsynaptic compartmentalization of the selected markers in PAZ, PSD and EXTRA fractions isolated from cortical synaptosomes derived from CB1-RS mice. (A) Representative Coomassie blue-stained SDS-PAGE and (B) Western blots carried out by immunoblotting increasing amounts of cortical synaptosomes (3, 6, 9 and 12 μg/lane) and different subsynaptic fractions of CB1-RS mice (3 μg/lane). Presynaptic fraction PAZ, postsynaptic fraction PSD and extrasynaptic fraction EXTRA. The efficiency of the subsynaptic separation was assessed using antibodies as markers of specific sub-cellular compartments: PSD fraction markers (PSD-95, Gephyrin, and Shank3) and EXTRA and PAZ fractions markers (Munc-18 and SNAP-25). Protein migration was consistent with their theoretical molecular mass, except for PSD-95 protein, which migrated at a molecular weight of 95 kDa (PSD 95, 80.4 kDa; Gephyrin, 83.2 kDa; SHANK, 185.3 kDa; Munc 18, 66.3 kDa; SNAP-25, 23.3 kDa). The molecular weights depicted correspond to the signal of the standard markers.

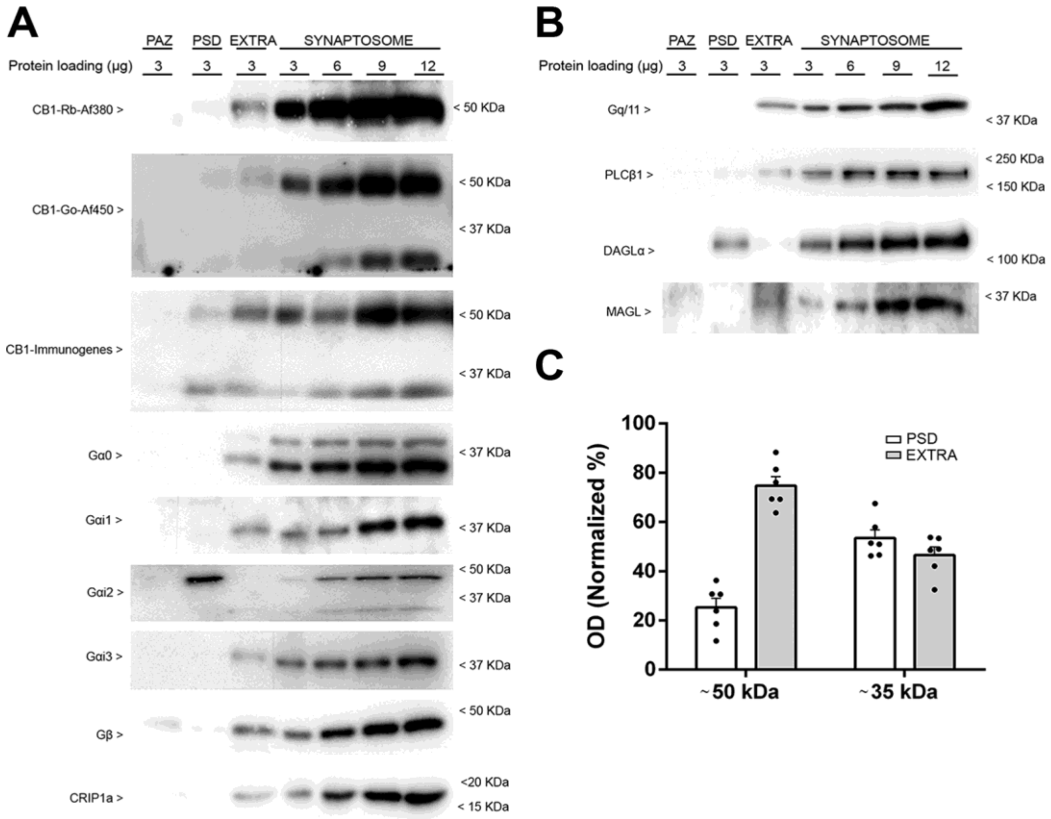

Figure S6. Subsynaptic compartmentalization of the CB1 receptor, the canonical transducers coupled to CB1 receptors, and other proteins of the endocannabinoid system in PAZ, PSD, and EXTRA fractions isolated from cortical synaptosomes derived from CB1-RS mice. Representative Western blots carried out by immunoblotting increasing amounts of cortical synaptosomes (3, 6, 9, and 12 µg/lane) and different subsynaptic fractions of CB1-RS mice (3 µg/lane) using antibodies against CB1 receptor, Gαi/o subtypes, Gβ and Crip1a (A) and Gαq/11, PLC-β1, DAGL-α, and MAGL (B). Presynaptic fraction PAZ, postsynaptic fraction PSD, and extrasynaptic fraction EXTRA. Protein migration was consistent with their theoretical molecular mass. For the CB1 receptor, Gαi2, and Gαo protein, extra bands migrating at ~ 35 kDa, ~ 36 kDa, and ~ 43 kDa were detected, respectively (CB1, 52.8 kDa; Gαo, 40.1 kDa; Gαi1, 40.5 kDa; Gαi2, 40.4 kDa; Gαi3, 40.5 kDa; Gβ (common), 37.3 kDa and 36.3 kDa 1 and 2 isoforms; CRIP1a, 18.6 kDa; Gαq/11, 42.0 kDa; PLC-β1, 138.3 kDa and 133.3 kDa, the β1a and β1b isoforms, respectively; DAGL-α, 115.3 kDa; MAGL, 33.3 kDa). The molecular weights depicted correspond to the signal of the standard markers. (C) The bar graphs show the subsynaptic distribution of the CB1 receptor immunoreactive signals of ~ 50 kDa and ~ 35 kDa bands obtained with the CB1-Immunogenes, CB1-Go-Af450 and CB1-Rb-Af380 antibodies. The quantification was performed using the data from the three antibodies together. The immunoreactive signals of PSD and EXTRA fractions are shown normalized to the total signal detected in both compartments. ~ 50 kDa: EXTRA 74.7 ± 3.6 vs. PSD 25.3 ± 3.6*; ~ 35 kDa: EXTRA 46.5 ± 3.2 vs. PSD 53.4 ± 3.2. Values correspond to the means ± SEM of six independent assays, using subsynaptic fraction preparations obtained from a pool of cortices of eight adult mice. Unpaired two tailed t test. * = $p < 0.05$.

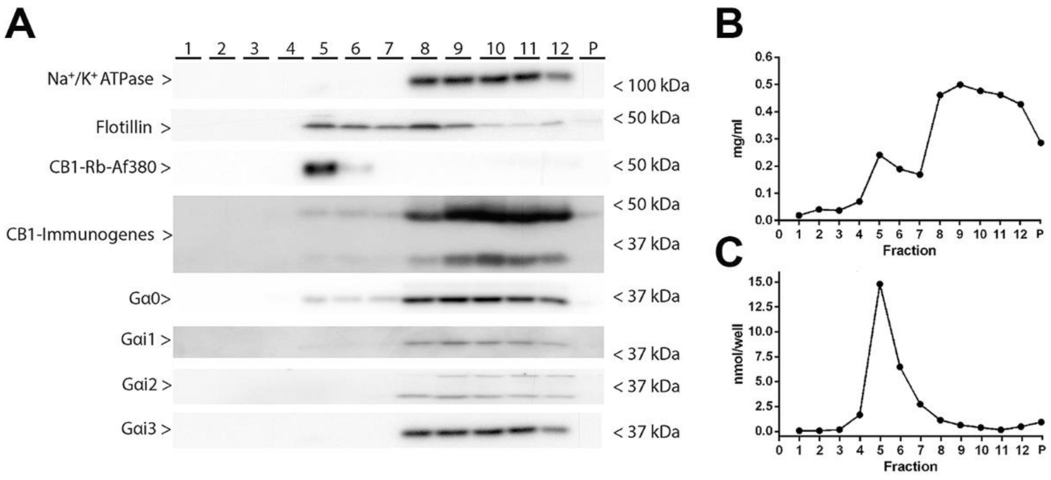

Figure S7. Alkaline phosphatase enzymatic activity, total protein amount and distribution of raft and non-raft markers in lipid raft and non-lipid raft fractions isolated from frontal cortical synaptosomes derived from CB1-RS mice. (A) Representative Western blots running in parallel same volume (20 µL/lane) of the collected 12 fractions and of the pellet (P). Immunoblot against Na^+/K^+-ATPase, Flotillin, CB1 receptor, and Gαi/o subtypes. Protein migration was consistent with their theoretical molecular mass. For the CB1 receptor and the Gαi2 protein, extra bands migrating at ~ 35 kDa and ~ 36 kDa were detected, respectively. Na^+/K^+-ATPase, 112.3 kDa; Flotillin, 47.5 kDa; CB1 receptor, 52.8 kDa; Gαo 40.1 kDa; Gαi1, 40.5 kDa; Gαi2, 40.4 kDa; Gαi3, 40.5 kDa. The molecular weights depicted correspond to the signal of the standard markers. (B) Total protein content of the collected 12 fractions and of the pellet (P). (C) Alkaline phosphatase activity of the collected 12 fractions and of the pellet (P).

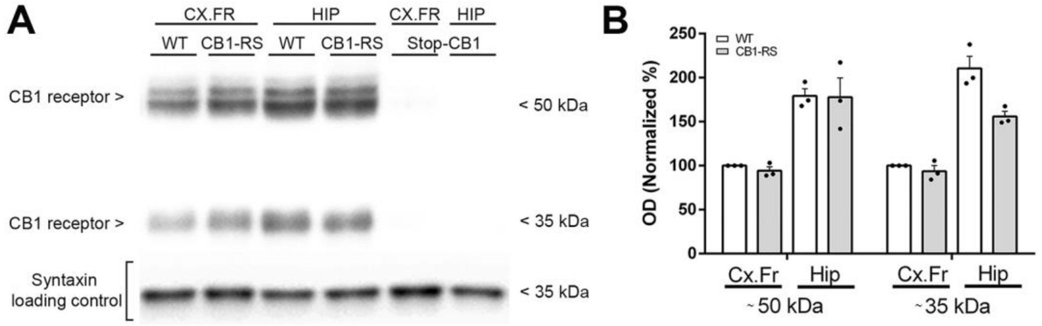

Figure S8. CB1 receptor protein expression in synaptosomes obtained from frontal cortical and hippocampal tissue of wild-type, CB1-RS and Stop-CB1 mice. (A) Representative image of Western blots carried out by immunoblotting same amounts of synaptosomes samples (20 µg protein/lane) using antibodies against CB1 receptor (CB1-Immunogenes) and syntaxin proteins, respectively. The molecular weights depicted correspond to the signal of the standard markers. Cx.Fr = Frontal cortex; Hip = Hippocampus; WT = Wild-type; CB1-RS = CB1 rescue; Stop-CB1 = Stop-CB1. (B) The graph shows the relative immunoreactivity values of ~ 50 kDa and ~ 35 kDa bands of the CB1 receptor, normalized to the signal of frontal cortical synaptosomes of wild-type mice.

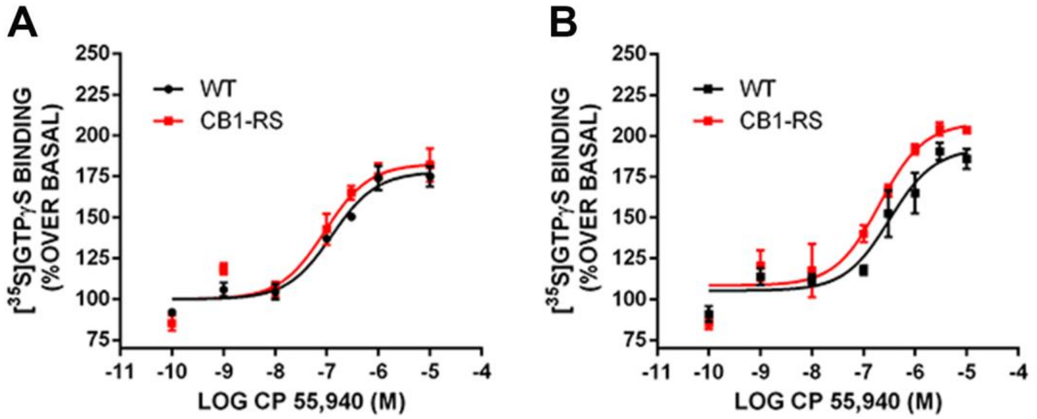

Figure S9. CB1 receptor coupling to Gαi/o proteins in synaptosomes obtained from frontal cortical and hippocampal tissue of wild-type and CB1-RS mice. CP 55,940-stimulated [^{35}S]GTPγS binding in frontal cortical synaptosomes (A) and in hippocampal synaptosomes (B). Concentration response curves were constructed using mean values ± SEM from triplicate data points of three independent experiments. Emax values are expressed as % specific [^{35}S]GTPγS bound of basal.

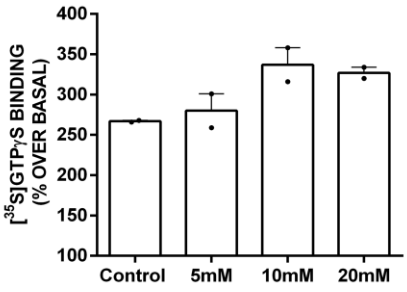

Figure S10. CB1 receptor coupling to Gαi/o proteins in control and in 5 mM, 10 mM and 20 mM MβCD pretreated synaptosomes from frontal cortical tissue of Glu-CB1-RS and GABA-CB1-RS mice. Bar graph of 10 μM CP 55,940 stimulated [^{35}S]GTPγS binding. Emax values are expressed as % specific [35S]GTPγS bound of basal.

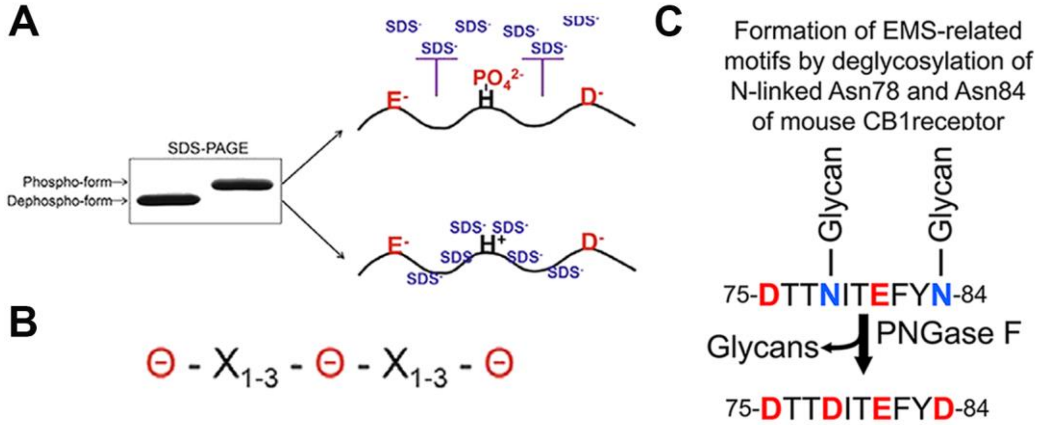

Figure S11. Model for the phosphorylation-dependent electrophoretic mobility shift (PDEMS) phenomenon and the EMS-related motif. Adapted from Figure 4 in Lee et al. (2019) [31]. (A) Molecular model for the PDEMS phenomenon. Protein phosphorylation induces the formation of the electrophoretic mobility shift (EMS)-related motif, which inhibits the binding of SDS to the peptide bond of proteins by charge–charge repulsion. Consequently, the decreased ratio of bound SDS per protein results in the mobility shift. (B) Consensus sequence of the EMS-related motif. Θ symbols corresponds to a negatively charged amino acid (E or D) or phosphorylated amino acid, and X

denotes any amino acid. (C). Appearance of two EMS-related motifs within the extracellular N-terminus of the mouse CB1 as a consequence of deamination of asparagines 78 and 84 following enzymatic deglycosylation using PNGase.

3. Supplementary Tables

Table S1. The list of primary antibodies used.

Antibody	Dilution IF	Dilution WB	Species & clonality	Immunizing antigen	Source, Cat. No.
CB1 receptor		1:1000	Goat polyclonal	Mouse CB1, C-terminal 31 aa (NM007726)	Frontier Institute CB1-Go-Af450
CB1 receptor		1:1250	Rabbit polyclonal	Mouse CB1, C-terminal 31 aa (NM007726)	Frontier Institute CB1-Rb-Af380
CB1 receptor		1:5000	Rabbit polyclonal	Repeated immunizations with a synthetic peptide corresponding to amino acid residues 443-473	Immunogenes anti-CB1 polyclonal antibody
DAGL-α		1:4000	Rabbit polyclonal	Mouse DGL-alpha, C-terminal 42 aa (NM198114)	Frontier Institute, DGL-Rb-AF380-1
Crip1a/b		1:500	Rabbit polyclonal	Peptide mapping within an internal region of CRIP1 of human origin.	Sta. Cruz Biotech. Inc sc-137401
Flotillin 1		1:1000	Rabbit polyclonal	Synthetic peptide conjugated to KLH derived from within residues 1 - 100 of Human Flotillin 1.	Abcam ab41927
Gαq/11		1:1000	Rabbit polyclonal	Peptide mapping within a domain common to Gα 11 of mouse origin.	Sta. Cruz Biotech. Inc sc-392
$G\alpha_o$		1:5000	Rabbit polyclonal	Peptide mapping within a highly divergent domain of Gαo of rat origin	Sta. Cruz Biotech. Inc sc-387
$G\alpha_{i1}$		1: 2500	Rabbit polyclonal	Peptide mapping within a highly divergent domain of Gαi-1 of rat origin	Sta. Cruz Biotech. Inc sc-391
$G\alpha_{i2}$		1: 1000	Rabbit polyclonal	Peptide mapping within a highly divergent domain of Gαi-2 of rat origin	Sta. Cruz Biotech. Inc sc-7276
$G\alpha_{i3}$		1:75000	Rabbit polyclonal	Peptide mapping at the C-Terminus of Gαi-3 of rat origin	Sta. Cruz Biotech. Inc Sc-262
Gβ		1:10000	Rabbit polyclonal	Peptide mapping at the C-terminus of Gβ of mouse origin	Sta. Cruz Biotech. Inc sc-378
GAPDH		1:2000	Mouse monoclonal	Not specified	Abcam ab8245
Gephyrin		1:1000	Rabbit polyclonal	Synthetic peptide conjugated to KLH derived from within residues 700 to the C-terminus of Mouse Gephyrin	Abcam, ab32206
GFAP	1:500	1:1000	Rabbit polyclonal	GFAP isolated from cow spinal cord	DAKO Z0334
Acetyl-Histone H3		1:1000	Rabbit monoclonal	Synthetic acetylated peptide corresponding to residues surrounding Lys9 of histone H3	Cell signaling, 9671
MAP2	1:500	-	Mouse monoclonal	Bovine MAP2.	Sigma-Aldrich M2320

MAGL		1:1000	Goat polyclonal	Synthetic peptide (C-QDLPHLVNADGQY) corresponding to internal sequence amino acids 17-29 of human MAGL	Abcam, ab77398
MUNC-18/STXBP1-IsoA		1: 5000	Goat polyclonal	Peptide C-SRVSFEDQAPTME from the C Terminus of NP_003156.1	Sigma-Aldrich; SAB2500659
Na^+/K^+ ATPase	-	1:450	Mouse monoclonal	α1 subunit of Na+/K+ ATPase purified from lamb kidney. The epitope maps at residues 496-506.	Sigma-Aldrich A277, clone M8-P1-A3
NMDAR1	-	1:2000	Rabbit monoclonal	Synthetic peptide corresponding to the C-terminus of rat NMDA receptor subunit (amino acids 909-938 rat NMDAR1)	Millipore AB9864
PLC-β_1 (N-ter)	-	1:750	Mouse monoclonal	Synthetic peptide corresponding to amino acids 4–159 of the rat PLCβ1.	BD Transduction Laboratories 610924
PSD95		1:5000	Rabbit polyclonal	Synthetic peptide corresponding to residues surrounding Gly99 of human PSD95	Cell Signaling 3450
Rab11		1:8000	Mouse monoclonal	Human Rab11 aa. 86-207	BD Transduction Laboratorie 610656
SHANK3		1:10000	Rabbit polyclonal	Synthetic peptide from SH3 region of Mouse SHANK3 conjugated to an immunogenic carrier protein	Abcam ab104702
SNAP-25	1:1000	1:4000	Mouse monoclonal	Tissue cell preparation: this antibody was raised against a crude synaptic preparation from the post mortem human brain.	Abcam, ab24732
Synaptophysin		1:8000	Rabbit polyclonal	Peptide corresponding to amino acids 41-62 of human synaptophysin	Abcam ab14692
Syntaxin		1:1000	Mouse monoclonal	Recombinant fragment corresponding to Rat Syntaxin aa 3-225	Abcam ab3265
CD90/Thy1		1:1000	Rabbit monoclonal	Synthetic peptide corresponding to residues near the N terminal of Human CD90 / Thy1 (UniProt ID: P04216)	Abcam, ab92574

Table S2. Migration profile of the CB1 receptor immunorreactive bands in cortical synaptosome samples obtained in the absence or the presence of protease inhibitors during the fractionation procedure. Values are means ± SEM of the ~ 35 kDa and ~ 50 kDa bands ratio (normalized to the condition where synaptosomes were obtained in the absence of protease inhibitors) obtained from three independent experiments. Unpaired two tailed t test; p = <0.05.

Synaptosomes obtained in the absence of protease inhibitors	Synaptosomes obtained in the presence of the protease inhibitors
1.00	1.13 ± 0.27

Table S3. Statistical analysis of the subsynaptic distribution of the CB1 receptor immunoreactive signals of ~ 50 kDa and ~ 35 kDa bands between CB1-RS and wild-type mice (WT). Values are means ± SEM of four independent assays, using preparations of subsynaptic fractions obtained

from a pool of cerebral cortices of eight adult mice. Unpaired two tailed t test. * = Statistical difference from WT; p = <0.05.

	WT ~ 50 kDa	CB1-RS ~ 50 kDa
**EXTRA**	67.9 ± 0.7	74.7 ± 3.6
**PSD**	32.0 ± 0.7	25.3 ± 3.6
	**WT ~35 kDa**	**CB1-RS ~35 kDa**
**EXTRA**	52.8 ± 3.9	46.5 ± 3.2
**PSD**	47.9 ± 4.8	53.4 ± 3.2

Table S4. Densitometry analysis of the specific immunorreactive signals (~ 50 kDa and ~ 35 kDa bands) of the CB1 receptor (see supplemental Figure S8), normalized to the signal of frontal cortical synaptosomes of wild-type. Values are means ± SEM of three independent experiments, using synaptosomes enriched preparations obtained from a pool of the frontal cortices and hippocampi of eight adult mice. Unpaired two-tailed t test. * = Statistical difference from WT; p = < 0.05.

~ 50 kDa	WT	CB1-RS
**Frontal cortex**	1.00	0.94 ± 0.05
**Hippocampus**	1.79 ± 0.08	1.77 ± 0.21
**~ 35 kDa**	**WT**	**CB1-RS**
**Frontal cortex**	1.00	0.93 ± 0.06
**Hippocampus**	2.10 ± 0.14	1.56 ± 0.06*

Table S5. Concentration response curves for the CP 55,940-stimulated specific [^{35}S]GTPγS binding in frontal cortical and hippocampal synaptosomes derived from wild-type (WT) and CB1-RS mice. Values are means ± SEM of three independent experiments, using synaptosomes enriched preparations obtained from a pool of the frontal cortices and hippocampi of eight adult mice. Unpaired two-tailed t test.

Frontal cortex	WT	CB1-RS
%E_{max}	190.7 ± 9.8	189.9 ± 3.6
pEC_{50}	6.88 ± 0.17	6.93 ± 0.06
Basal (cpm)	14,548 ± 1,788	20,765 ± 2,484
**Hippocampus**	**WT**	**CB1-RS**
%E_{max}	223.3 ± 22.85	229.6 ± 15.91
pEC_{50}	6.37 ± 0.25	6.74 ± 0.04
Basal (cpm)	20,013 ± 487	17,051 ± 409

Table S6. CB1 receptor protein expression in synaptosomal membranes of frontal cortex and hippocampus by the slope comparison method of the lines obtained by regression analysis of the data shown in Figure 5B-D. Values are the slopes ratio obtained from five and seven independent experiments carried out in frontal cortex and hippocampus, respectively. Both in frontal cortex and hippocampus, the experiments were carried out using two preparations enriched in synaptosomes, each of them obtained from pools of the frontal cortices and hippocampi of eight adult mice. * = statistically significant differences; $p < 0.05$.

**Frontal cortex**			
**Slopes ratio**	**Glu-CB1-RS / CB1-RS**	**GABA-CB1-RS / CB1-RS**	**Glu-CB1-RS / GABA-CB1-RS**
**~ 50 kDa**	0.44*	0.42*	1.04
**~ 35 kDa**	0.49*	0.42*	1.15
**Hippocampus**			
**Slopes ratio**	**Glu-CB1-RS / CB1-RS**	**GABA-CB1-RS / CB1-RS**	**Glu-CB1-RS / GABA-CB1-RS**
**~ 50 kDa**	0.28*	0.71*	0.40*
**~ 35 kDa**	0.33*	0.63*	0.53*

Table S7. Stimulation of [^{35}S]GTPγS binding by a maximal concentration (10 μM) of the cannabinoid agonist CP 55,940 in cortical synaptosomes from wild-type mice pretreated with MβCD, as described in Section 4.7 from Materials and Methods. Values are means ± SEM of two independent experiments performed in duplicate, using synaptosomes enriched preparations obtained from a pool of the frontal cortices of eight adult mice.

	**Control**	**5 mM MβCD**	**10 mM MβCD**	**20 mM MβCD**
%E_{max}	267 ± 10	280 ± 21	337 ± 21	327 ± 07
**Basal (cpm)**	8,959 ± 2143	9,456 ± 1426	8,227 ± 1228	7,574 ± 388

Table S8. Stimulation of [^{35}S]GTPγS binding by a maximal concentration (10 μM) of the cannabinoid agonists CP 55,940 and WIN 55,212-2 in cortical synaptosomes from Glu-CB1-RS and GABA-CB1-RS mice pretreated with MβCD (10 mM), as described in Section 4.7. from Materials and Methods. Values are means ± SEM of three independent experiments performed in triplicate, using synaptosomes enriched preparations obtained from a pool of the eight adult mice. Unpaired (%E_{max}) or paired (Basal) two-tailed t test. * = statistically significant over control, $p < 0.05$.

	**Glu-CB1-RS**		**GABA-CB1-RS**	
	**Control**	**MβCD**	**Control**	**MβCD**
**%E_{max} CP 55,940**	177.33 ± 4.73	225.87 ± 8.75*	189.2 ± 1.10	255 ± 7.76*
**%E_{max} WIN 55,212-2**	90.37 ± 4.72	140.50 ±13.02*	105.20 ± 3.97	158.30 ± 8.42*
Basal (cpm)	22,081 ± 1,791	15,397 ± 1,578*	22,938 ± 5,137	16,384 ± 182

Table S9. Summary table of the CB1 receptor density and the CB1 receptor coupling to Gαi/o in cortical and hippocampal synaptosomes from wild-type (WT), CB1-RS, Glu-CB1-RS, and GABA-CB1-RS mice.

	**Frontal cortical synaptosomes**				**Hippocampal synaptosomes**			
	**WT**	**CB1-RS**	**Glu-CB1-RS**	**GABA-CB1-RS**	**WT**	**CB1-RS**	**Glu-CB1-RS**	**GABA-CB1-RS**
**CB1 receptor density**	++	++	+	+	++++	++++	+	+++
**CB1 receptor-coupling Gαi/o**	++	++	+	+	++++	++++	+	+++

	**Frontal cortical synaptosomes**					**Subsynaptic distribution in cortical synaptosomes**					
	**Control**		**MβCD**			**WT**			**CB1-RS**		
	**Glu-CB1-RS**	**GABA-CB1-RS**	**Glu-CB1-RS**	**GABA-CB1-RS**		**PAZ**	**PSD**	**EXTRA**	**PAZ**	**PSD**	**EXTRA**
**CB1 receptor-Gαi/o coupling**	+	+	++	++	**CB1 receptor density**		+	++		+	++

The lipid-raft vs. non-lipid raft targeting of the CB1 receptor was qualitatively indistinguishable between wild-type and CB1-RS**.** Note: The symbols represent the relative levels of the CB1 receptor density and the CB1 receptor coupling to Gαi/o between mice lines, determined by Western blot and agonist-stimulated [^{35}S]GTPγS binding assays, respectively: ++++, very high ; +++, high; ++, moderate; +, low; These data are for illustration and must be considered informative. Quantitative data are depicted through tables or figures legends in the main text and in the supplemental material.

References

31. Lee, C.R.; Park, Y.H.; Min, H.; Kim, Y.R.; Seok, Y.J. Determination of protein phosphorylation by polyacrylamide gel electrophoresis. J. Microbiol. 2019, 57, 93–100, doi:10.1007/s12275-019-9021-y.
65. Fleming, W.W.; Westfall, D.P.; De la Lande, I.S.; Jellett, L.B. Log-normal distribution of equiefective doses of norepinephrine and acetylcholine in several tissues. *J. Pharmacol. Exp. Ther.* **1972**, *181*, 339–45.
66. Christopoulos, A. Assessing the distribution of parameters in models of ligand–receptor interaction: to log or not to log. *Trends Pharmacol. Sci.* **1998**, *19*, 351–357, doi:10.1016/S0165-6147(98)01240-1.
